# Influenza vaccination coverage in Chiburijima Island, Japan: Impact of diversification of vaccination place

**DOI:** 10.1002/jgf2.335

**Published:** 2020-05-27

**Authors:** Takuya Itamochi, Makiko Mieno, Shuji Hatakeyama

**Affiliations:** ^1^ Ohchi Municipal Hospital Shimane Japan; ^2^ Department of Medical Informatics Center for Information Jichi Medical University Tochigi Japan; ^3^ Division of General Internal Medicine/Infectious Diseases Jichi Medical University Hospital Tochigi Japan

**Keywords:** health promotion, influenza vaccine, remote island medicine, vaccination place, vaccination rate

## Abstract

**Background:**

To increase the influenza vaccination rate in Chiburijima Island, we diversified vaccination places from a single clinic to the seven town halls from all the districts.

**Methods:**

We retrospectively analyzed influenza vaccination coverage, vaccinees' district of residence, and location of vaccination place, from prevaccination screening questionnaires between October 2007 and March 2018. Except for home‐visiting vaccination services, before 2011, influenza vaccination was provided only at a single clinic; after 2012, it was provided at the seven town halls in addition to the clinic. We calculated the vaccination rates among residents of Chiburijima Island, stratified by age groups and districts of residence.

**Results:**

Estimated influenza vaccination rates for all ages increased from 38% in 2007 to 58% in 2017. There was a 14% increase in coverage in 2010 (the year following the 2009 H1N1 pandemic) and a slight increasing trend after 2012. The vaccination coverage of residents, particularly of people ≥65 years old and living >1 km from the former vaccination place, increased after diversification of the vaccination place.

**Conclusions:**

Influenza vaccination rate in Chiburijima Island increased by 20% between 2007 and 2017, although the rate among the elderly Japanese people decreased by 4% based on the national data of routine immunization. Shortening the distance to vaccination places might improve the coverage as elderly people are often restricted due to transportation means. Sustained and multifaceted strategies are necessary for better immunization coverage.

## Graphical abstract

Influenza vaccination rate in Chiburijima Island increased by 20% between 2007 and 2017, although the rate among the elderly Japanese people decreased by 4% based on the national data of routine immunization. Shortening the distance to vaccination places might significantly improve the coverage as elderly people are often restricted due to transportation means.

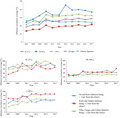

## 1 INTRODUCTION

Vaccination is one of the important measures to control influenza. A meta‐analysis showed that pooled efficacy of influenza trivalent vaccine was 59% in adults aged 18‐65 years.^1^ In another meta‐analysis conducted in 2018, elderly people who received the vaccine experienced influenza less frequently than the placebo group (risk ratio, 0.42).^2^ Although controversy exists on the effect of the vaccination on mortality among the elderly, there is a study showing that influenza vaccine for elderly people reduces the rate of hospitalization and deaths associated with influenza.^3^ Due to herd immunity, vaccinating most schoolchildren causes a reduction in mortality from influenza among the elderly.^4^ Increasing vaccine access (eg, convenient timings and places of vaccination, reducing waiting time, and ensuring prompt delivery of vaccines), educating people, advocating vaccination, garnering political and financial support, and overcoming immunization‐related barriers have been recognized as potential factors associated with improving influenza vaccination coverage.^5^, ^6^

Chiburijima Island, one of the Oki Islands UNESCO Global Geopark (http://www.oki-geopark.jp/en/features/), is an isolated island belonging to Shimane prefecture, Japan, and is located in the Sea of Japan, approximately 50 km north of Shimane Peninsula. The land area is 13 km^2^ with a population of approximately 600 as of 2015. Chiburijima Island is currently divided into 7 districts: Oe, Kori, Kurii, Tataku, Nibu, Usuge, and Urumi. Oe district is the central part of the village, where the clinic, schools, and a majority of workplaces are located. There is only a single medical facility (clinic/doctor's office) with a full‐time doctor. To increase influenza vaccination coverage, we changed the vaccination place from the clinic to public places of each district, in October 2012. In this study, using prevaccination screening questionnaires, we investigated the influenza vaccination rate in this island between 2007 and 2018 and assessed the effect of diversification of vaccination place on vaccination coverage.

## 2 METHODS

To analyze influenza vaccination coverage in Chiburijima Island, we conducted a retrospective study using prevaccination screening questionnaires between October 2007 and March 2018. Vaccinees' age, gender, residence address (the zoning district in the island), vaccination place, and vaccination date were extracted from the questionnaire, which was stored in Chibu Village Clinic.

Between October 2007 and March 2012, influenza vaccination was provided at a single institute (the clinic/doctor's office located in Oe). Between October 2012 and March 2018, expecting to enhance the coverage, influenza vaccination was provided at each town (public) hall in the seven districts, in addition to the clinic in Oe. Visiting vaccination services at a nursing home and patients' homes were also available throughout the study period.

We calculated vaccination rates stratified by age group (0‐15 years, 16‐64 years, and 65 years and older) and district of residence. The number of vaccination was counted only once if the second dose of influenza vaccine was used in the same season. Estimated population of the districts of the island, according to the Basic Resident Register of Chibu village, Oki County, Shimane prefecture, Japan, was used as a denominator.

Furthermore, we also compared the vaccination coverage of residents of Chiburijima Island with that of elderly Japanese people in general. In Japan, after 2001, annual influenza vaccination is recommended with financial support provided by the local governments for people aged 65 years and older, and for people aged 60‐64 years who have certain chronic illnesses or are immunosuppressed. Therefore, we adopted the national data for elderly vaccination^7^ to compare vaccination coverage trends.

To evaluate the impact of demographic change of vaccination place, annual influenza vaccination coverage was also compared with the distance from residential location to the clinic (Oe district), which was the only vaccination place before March 2012. The distances from the clinic to each town hall were as follows: 0.1 km in Oe, 0.5 km in Kori, 1.2 km in Kurii, 1.2 km in Tataku, 2.2 km in Nibu, 2.7 km in Usuge, and 3.9 km in Urumi. In the island, public transportation is not available except for a bus and taxis. There is only a single one‐way bus each day that loops around the village; the route is village office—town hall in each district—clinic—village office, with the journey taking approximately 60 min. Most families have private cars or motorcycles and usually use them for long‐distance transportation instead of taxis. For short‐distance transportation (eg, home to town hall, home in Oe or Kori to the clinic), people usually get there by walking or bicycling.

If two doses of influenza vaccine were used in the same season, the district where the first vaccination had taken place was adopted in this study. There are single kindergarten, elementary school, and junior high school in the island; all of them are located in Oe district near the clinic. Therefore, in principle, children aged under 15 years were recommended to receive the influenza vaccine in Oe district throughout the study period. Residents aged 16 years and older were recommended to receive the vaccine at the clinic (in Oe district) before March 2012 and at the town hall in the district of their residence, after October 2012. In each district other than Oe, we provided influenza vaccination in one day of the influenza season. In Oe district, we provided influenza vaccination on two days approximately a month apart, because children under 13 years occasionally received influenza vaccine twice a year according to Japanese guidelines.^8^ For people who could not meet the schedule, vaccinations were provided at the clinic.

This study was approved by the Ethics Committee of the Shimane Prefectural Central Hospital (Number R18‐045). Written informed consent from the patients enrolled was waived by the Ethics Committee because of the retrospective design of the study.

## 3 RESULTS

**FIGURE 1:**
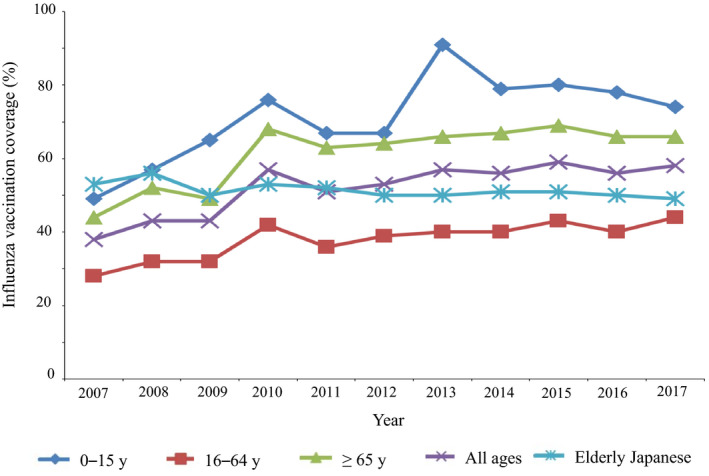
Estimated influenza vaccination coverage among residents in Chiburijima Island and elderly Japanese individuals aged 65 y and older and those aged 60‐64 y with certain chronic illnesses or are immunocompromised. The vaccination rates among elderly Japanese were estimated according to the national data of routine immunization

The population trends between 2007 and 2017 by age group (0‐15 years, 16‐64 years, and ≥65 years) and districts (Oe, Kori, Kurii, Tataku, Nibu, Usuge, and Urumi) of Chiburijima Island are shown in the Table 1. Estimated influenza vaccination coverage for all ages in Chiburijima Island increased from 38% in 2007 to 58% in 2017 (Figure 1). There was a 14% increase of coverage in 2010 (the year following the 2009 H1N1 pandemic) and a slight increasing trend after 2012 (the year the vaccination place diversified). The vaccination rate in 2010 was significantly higher compared with the rate in 2007 (57.3% vs 37.5%, *P* < .0001 by the chi‐squared test).

**TABLE 1:** Population trends between 2007 and 2017 by age group and districts in Chiburijima Island

Year and age group	Number of population (people) in each district							
	Oe	Kori	Kurii	Tataku	Nibu	Usuge	Urumi	Total
2007								
0‐15 y	11	9	19	16	13	15	0	83
16‐64 y	29	86	42	39	81	42	11	330
≥65 y	44	68	23	62	62	25	20	304
All ages	84	163	84	117	156	82	31	717
2008								
0‐15 y	11	8	12	18	11	15	0	75
16‐64 y	29	82	36	37	76	35	9	304
≥65 y	42	61	22	64	59	28	22	298
All ages	82	151	70	119	146	78	31	677
2009								
0‐15 y	12	8	15	17	12	13	0	77
16‐64 y	27	77	35	38	76	33	10	296
≥65 y	39	61	21	63	57	31	20	292
All ages	78	146	71	118	145	77	30	665
2010								
0‐15 y	12	7	14	18	9	14	0	74
16‐64 y	29	79	32	36	74	28	7	285
≥65 y	37	58	20	62	60	31	20	288
All ages	78	144	66	116	143	73	27	647
2011								
0‐15 y	13	7	14	6	11	14	2	67
16‐64 y	27	77	33	33	73	27	9	279
≥65 y	32	56	18	59	59	33	21	278
All ages	72	140	65	98	143	74	32	624
2012								
0‐15 y	6	7	15	6	5	17	2	58
16‐64 y	23	69	31	31	72	31	6	263
≥65 y	30	58	17	58	61	32	21	277
All ages	59	134	63	95	138	80	29	598
2013								
0‐15 y	5	7	13	6	6	14	2	53
16‐64 y	20	72	33	27	68	33	6	259
≥65 y	28	60	18	58	66	33	20	283
All ages	53	139	64	91	140	80	28	595
2014								
0‐15 y	4	9	16	4	7	14	2	56
16‐64 y	20	68	42	24	64	32	6	256
≥65 y	23	64	19	56	65	34	21	282
All ages	47	141	77	84	136	80	29	594
2015								
0‐15 y	5	9	19	4	7	14	2	60
16‐64 y	21	64	45	27	56	30	6	249
≥65 y	25	63	19	53	72	35	19	286
All ages	51	136	83	84	135	79	27	595
2016								
0‐15 y	5	10	21	4	9	12	2	63
16‐64 y	25	65	48	28	60	30	5	261
≥65 y	25	63	21	50	77	35	19	290
All ages	55	138	90	82	146	77	26	614
2017								
0‐15 y	12	12	21	4	9	11	3	72
16‐64 y	29	64	45	26	52	30	12	258
≥65 y	26	64	23	47	73	33	19	285
All ages	67	140	89	77	134	74	34	615

In contrast, the vaccination rates among elderly Japanese, based on the national data of routine immunization^7^, showed a temporary increase in 2010, but then decreased and remained stable (53% in 2010 and 49% in 2017) (Figure 1). Until 2009, the vaccination coverage in people aged 65 years and older in Chiburijima Island was lower than that of the elderly Japanese people who received routine influenza vaccination, but the vaccination rates among the elderly in Chiburijima Island became higher after 2010.

**FIGURE 2:**
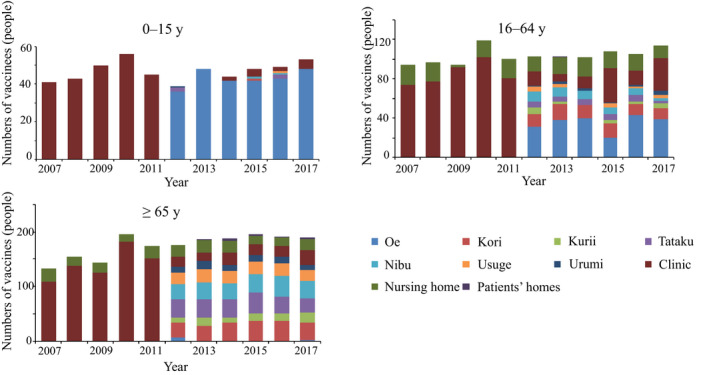
Trends of number of people who received influenza vaccines stratified by age group and vaccination places. Before 2011, influenza vaccination was basically provided at the clinic. After 2012, people were advised to receive vaccines at the town halls in the districts of their residence

**FIGURE 3:**
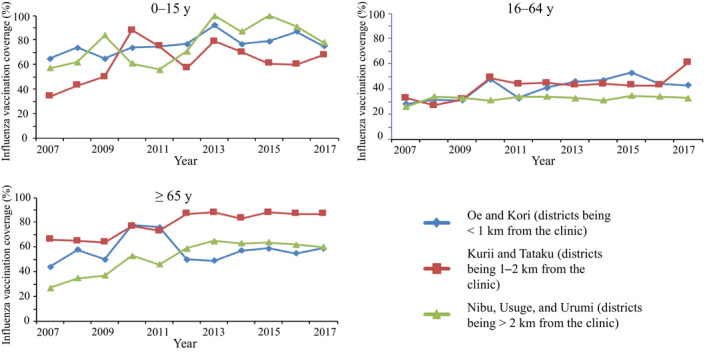
Trends of influenza vaccination rates stratified by districts of residence and age group. The distances from the clinic to town halls in each district were 0.1 km in Oe district, 0.5 km in Kori, 1.2 km in Kurii, 1.2 km in Tataku, 2.2 km in Nibu, 2.7 km in Usuge, and 3.9 km in Urumi

The numbers of people who received influenza vaccination at each place stratified by age group are shown in Figure 2. After 2012, among people aged 65 years and older, more than 80% were vaccinated at the respective town halls of the seven districts where they resided. In contrast, people aged 16‐64 years more frequently received vaccination at Oe district. Figure 3 shows the trend of vaccination rate by age group and distance between vaccination place and the clinic. In the age group of ≥65 years, people living in the remotest district from the clinic had the lowest vaccination coverage before 2011. However, there was a 14% and 13% increase of coverage in 2012, in districts located at 1‐2 km (Kurii and Tataku) and ≥2 km (Nibu, Usuge, and Urumi), respectively, from the clinic. In contrast, the vaccination rate in districts <1 km from the clinic (Oe and Kori) after 2012 showed similar levels to those before 2009. In the age group of <65 years, similar vaccination rates were maintained between 2012 and 2017.

## 4 DISCUSSION

In this study, the 2009 influenza pandemic had a strong impact on increasing the vaccination rate in all age groups (14% rise in 2010). Pandemic 2009 H1N1 influenza vaccination was implemented in a separate frame around the same time as seasonal influenza vaccination in 2009. Since many people received the 2009 H1N1 influenza vaccine, the number of people who received seasonal influenza vaccine probably did not increase simultaneously in 2009. Although influenza vaccination coverage temporarily rebounded (6% drop) in all age groups in 2011, it gradually increased again after 2012, the year the vaccination place diversified.

The majority of people aged 0‐15 years received influenza vaccination in Oe district after 2012, because the kindergarten, elementary school, and junior high school are located there. As a result, the vaccination rate of this cohort increased after diversifying the vaccination place in 2012. In the age group of 16‐64 years, the vaccination rate remained relatively stable after diversification of the vaccination place in 2012. The diversification of vaccination place may not influence the working population, as they have more sufficient means of transportation than the elderly. Since many work places are in Oe, a certain number of workers would get vaccinated at Oe district, rather than the districts where they resided. In addition, there may be other strong factors influencing immunization coverage in this highly mobile generation, such as low consciousness of own health, financial problems, and time pressure due to work.^5^, ^6^, ^9^

In the age group of ≥65 years, the vaccination coverage of residents living at a distance (more than 1 km) from the clinic (the only vaccination place before 2011) increased after diversification of the vaccination place. Since the elderly are often restricted due to transportation issues, diversification of the vaccination place provides opportunities for vaccination in their neighborhood, thereby playing an important role in increasing vaccination coverage in this study. It was reported that distance and transportation problems were the barriers to seeking health care among Japanese elderly people.^10^

Meanwhile, approximately a 25% drop in the vaccination rate of the elderly people living in Oe and Kori (<1 km distant from the clinic) was observed after 2012. One possible explanation is that the waiting time during the vaccination visit was probably prolonged in Oe district after 2012 because more than 25 people per hour including schoolchildren were vaccinated at the same place; as a result, elderly people in Oe may have hesitated to get the influenza vaccination. Although we had no correct data on the waiting time, the mean numbers of vaccinees per hour between 2012 and 2017 were 27.3 in Oe, 22.9 in Kori, 8.7 in Kurii, 19.3 in Tataku, 19.7 in Nibu, 12.7 in Usuge, and 6.9 in Urumi. As the number of medical staff was the same in each vaccination place, the waiting time for vaccination in Oe district was probably longer in other districts. It is known that reducing waiting time is one of the important strategies for increasing influenza vaccination coverage.^5^

Although the vaccination rates among the elderly in Japan, based on the national data according to the national routine immunization policy,^7^ slightly decreased (from 53% in 2010 to 49% in 2017), an increasing trend of influenza vaccination coverage among people aged ≥65 years in Chiburijima Island was noted after 2012. This may imply that measures, including diversification of the vaccination place, to shrink the distance to the vaccination place in 2012, would have some effect on increasing the vaccination rates in the Island, especially for elderly people with limited mobility. In addition, it may facilitate a vaccination propaganda within the community. School promotions and public relations in each district, regarding vaccination date, time, and place, might facilitate intradistrict (intraregional) coordination of influenza vaccination.

A combination rather than a single measure is important to improve vaccination coverage.^5^, ^6^ Reports also exist that showed the effects of weekend inoculation, education, information provision, and sending short message services through mobile phones, in increasing the vaccination rate.^11^, ^12^

This study has several limitations. This is an observational study in a remote island with a small population and limited number of healthcare workers. In addition, it is difficult to vaccinate in places other than medical facilities, such as town halls, unless there is a provision for handling medical emergencies (adverse events), including anaphylaxis, following vaccination. Therefore, the results of this study are probably not generalizable.

In conclusion, the influenza vaccination rate in Chiburijima Island increased by 20% between 2007 and 2017 with a 14% increase in coverage in 2010, the year following the 2009 H1N1 pandemic, with a slight increasing trend after diversification of the vaccination place in 2012. A greater increase of vaccination rate after changing vaccination place was observed in elderly people living far from the clinic (formerly the only vaccination place). Sustained approaches are warranted to improve vaccination coverage in the community.

## CONFLICT OF INTEREST

The authors have stated explicitly that there are no conflicts of interest in connection with this article.
